# Characterization of DJ-1, PTEN, and p-Akt as Prognostic Biomarkers in the Progression of Oral Squamous Cell Carcinoma

**DOI:** 10.7759/cureus.34436

**Published:** 2023-01-31

**Authors:** Mourad Kerdjoudj, Rey A De La Torre, Hilal Arnouk

**Affiliations:** 1 Internal Medicine, Chicago College of Osteopathic Medicine, Midwestern University, Downers Grove, USA; 2 Osteopathic Medicine, Arizona College of Osteopathic Medicine, Midwestern University, Glendale, USA; 3 Pathology, Chicago College of Osteopathic Medicine, Midwestern University, Downers Grove, USA; 4 Pathology, College of Graduate Studies, Midwestern University, Downers Grove, USA; 5 Pathology, College of Dental Medicine-Illinois, Midwestern University, Downers Grove, USA; 6 Pathology, Chicago College of Optometry, Midwestern University, Downers Grove, USA; 7 Molecular Pathology, Precision Medicine Program, Midwestern University, Downers Grove, USA

**Keywords:** metastatic cancer, oral epithelial dysplasia, p-akt, pten, dj-1, prognostic biomarker, diagnostic biomarkers, tumor progression, oral squamous cell carcinoma, head and neck cancer

## Abstract

Objective

Oral cancer has a five-year survival rate of 68%, and the methods used to assess it still rely heavily on morphology. Protein biomarkers can potentially increase the predictive power of histopathological evaluation. This study aims to examine the expression of three closely linked proteins implicated in the pathogenesis of oral squamous cell carcinoma (OSCC); protein deglycase (DJ-1), an oncogene, phosphatase, and tensin homolog (PTEN), a tumor suppressor gene, and phosphorylated protein kinase B (p-Akt), the activated form of a vital serine/threonine kinase, which is involved in the oncogenesis of several human malignancies, throughout the tumor progression steps to establish their potential as prognostic biomarkers.

Study design

Western blot analysis was carried out using four different cell lines representing the successive steps of OSCC progression, including normal oral keratinocytes, dysplastic oral keratinocytes, locally invasive OSCC, and metastatic OSCC.

Results

DJ-1 expression was found to be upregulated gradually throughout the successive steps of OSCC progression from normal to dysplastic to locally invasive to metastatic OSCC. PTEN expression showed an overall opposite trend. Interestingly, a significant downregulation of p-Akt was seen in the locally invasive OSCC cells, although it was followed by a significant increase in p-Akt expression in the metastatic OSCC cell line, which is consistent with the role of p-Akt in the motility and migration of cancer cells.

Conclusion

This study documented trends in expression patterns of three important signaling molecules, DJ-1, PTEN, and p-Akt, in normal, premalignant, and malignant oral keratinocytes. The oncogenic DJ-1 and tumor suppressor PTEN were expressed in a manner consistent with their respective roles in tumorigenesis, while p-Akt only showed a significant upregulation in the metastatic OSCC cells. Overall, all three proteins exhibited unique trends throughout the progressive stages of OSCC tumor progression, thereby adding to their potential utility as prognostic biomarkers for oral cancer patients.

## Introduction

Oral cancer ranks as the seventh most frequent type of cancer and the ninth most common cause of death across cancers worldwide [1]. In terms of proportionality, it accounts for 2%-4% of all cancers worldwide, with some regions demonstrating higher prevalence, such as India and Pakistan, at 45% and 10%, respectively [2]. Oral squamous cell carcinoma (OSCC) is a subgroup of cancers classified as head and neck cancer and is defined as cancer affecting the mucosal layer in the oral cavity and pharynx. This makes up 90% of all head and neck cancers. The two major risk factors for OSCC are alcohol consumption and tobacco use [3]. The current relative five-year survival rate for oral and pharyngeal cancer is 68% [4]. Patients typically have higher survival rates when the cancer is caught prior to spreading beyond the site of origin; however, OSCC is usually diagnosed in later stages due to misdiagnosis by the physician or from failure to notice the lesion by the patient [2,5].

The methods currently employed to assess OSCC prognosis rely on TNM classification and histopathological grading [6]. Both of these prognostic tools have been critically examined [7], and it has been found that there are some shortcomings regarding their ability to predict the outcome of cancer [8]. For this reason, newer methods are being evaluated to help augment the current methods in place. These newer methods include utilizing predictive protein biomarkers that can better assess tumorigenesis.

Molecular biomarkers can provide great utility in the management of cancer treatment, with applications ranging from screening, risk assessment, and determination of prognosis, among many others [9]. Hence, the purpose of this study is to assess whether there is any quantitative significance pertaining to the expression of certain biomarkers among the various stages of OSCC. The biomarkers of interest for this study are DJ-1, PTEN, and p-Akt.

DJ-1 is a protein that belongs to the ThiJ/PfpI/DJ-1 superfamily and is mapped to chromosome 1p36 [10]. Also known by the alternative name PARK-7, it has been identified as an oncogene that is upregulated in several types of cancer [11]. One of the ways by which it is thought to be involved in cancer progression is by its ability to serve as an inhibitor to the tumor suppressor PTEN [12]. Studies have shown that DJ-1 inhibits PTEN expression, causing subsequent tumor cell proliferation and migration [13,14]. Genetic expression of this protein is upregulated in a variety of cancers, and it has been shown that its overexpression correlates with poor prognosis in glottic and supraglottic squamous cell carcinoma (GSCC, SCCC) [10,12]. It has also been shown that targeting DJ-1 with siRNA can effectively inhibit DJ-1 expression, which subsequently enhances apoptosis and reduces the proliferation of cancerous cells [10]. This essentially makes it an attractive target for treatment. Understanding the relationship between the expression of DJ-1 and the various stages of cancer progression could thus help provide more insight into this protein’s utility as a prognostic marker for OSCC.

PTEN is a phosphatase that plays an important role in the PI3K/Akt signaling pathway of cell survival [15]. PTEN serves as a tumor suppressor, and its downregulation has been associated with a variety of different cancers and disease states [16,17]. PTEN plays a critical role in halting the signaling cascade that leads to increased cell survival and growth. It does this by dephosphorylating phosphatidylinositol-3, 4, 5-trisphosphate (PIP3) to phosphatidylinositol-4, 5-bisphosphate (PIP2), which, in turn, inhibits the activation of phosphoinositide-dependent kinase-1 (PDK-1). This, in turn, decreases the phosphorylation of Akt, thereby inhibiting it and leading to decreased overall cell survival and growth [15]. PTEN itself can be regulated at the transcriptional level as well as through several post-translational modifications [18].

p-Akt is the activated form of Akt, which is a serine/threonine kinase that is activated by several receptor tyrosine kinases and is involved in the Akt/PI3K/PTEN pathway. Its role is involved in the signaling cascade downstream of phosphatidylinositol 3-kinase (PI 3-kinase) and PDK-1, after which, once phosphorylated by PDK-1, it ultimately leads to increased cell survival and growth [19]. Overexpression of p-Akt has been associated with metastasis in non-small cell lung carcinoma (NSCLC) as well as poor prognosis in NSCLC, gastric cancer, breast cancer, and diffuse large B-cell lymphoma [20-23]. Considering its role in a pathway essential to cell growth and proliferation and its relationship to poor prognosis, p-Akt is an important molecule to consider if we are to assess the relationship between the other two biomarkers and the steps of tumorigenesis.

The relationship amongst the three proteins of interest and their role in cancer cell survival, proliferation, invasion, and migration makes them potentially useful molecular biomarkers and therapeutic targets for OSCC. The overall goal of this study was to assess the utility of the DJ-1, PTEN, and p-Akt as prognostic indicators in the progression of OSCC via the analysis of cellular protein expression across several cell lines representing normal gingival keratinocytes (PKG), dysplastic oral keratinocytes (DOK), locally invasive oral cancer (SCC25), and metastatic oral cancer (Detroit 562). We predicted that DJ-1 and p-Akt expression would show a stepwise increase from normal to dysplastic to locally invasive to metastatic cell lines, while PTEN would show an opposite trend along the successive steps of tumor progression.

## Materials and methods

Cell culture

A total of four different human cell lines were used to model the different stages of OSCC progression. Specifically primary gingival keratinocytes (PGK) were used to model normal oral squamous cells, dysplastic oral keratinocytes (DOK) that were originally isolated from a lesion with a mild to moderate dysplasia in the dorsal tongue of a 57-year-old man after a squamous-cell carcinoma was removed, representing premalignant oral lesions such as leukoplakia, SCC25 representing locally invasive squamous cell carcinoma cells, and Detroit 562 representing metastatic oropharyngeal cancerous cells. SCC25, Detroit 562, and primary gingival epithelial cells were acquired from ATCC (Manassas, VA), and DOK cells were acquired from the European Collection of Authenticated Cell Cultures (ECACC) (Salisbury, United Kingdom) via Millipore Sigma (Darmstadt, Germany). All cell lines were cultured and maintained in their respective specialized media according to providers’ recommendations.

Western blotting

DJ-1, PTEN, p-Akt, and total-Akt expression were quantitatively assessed in the four cell lines representing the primary stages of OSCC progression. The cells were harvested, lysed, and the resulting samples were loaded equally onto a 4%-20% Tris-glycine sodium dodecyl sulfate (SDS) polyacrylamide gel electrophoresis. Following the electrophoresis, cellular proteins were transferred onto a polyvinylidene fluoride (PVDF) membrane. The membranes were subsequently blocked in 5% nonfat milk, then incubated overnight at 4°C in 5% nonfat milk containing working dilutions of primary antibodies as follows: 1:2000 for DJ-1 antibody (Catalog number 11681-1-AP, Proteintech, Rosemont, IL), 1:2000 for PTEN antibody (Catalog number 9559, Cell Signaling, Danvers, MA), 1:2000 for p-Akt antibody (Catalog number 9271, Cell Signaling, Danvers, MA), 1:2000 for total-Akt antibody (Catalog number 4691, Cell Signaling, Danvers, MA), and 1:10000 for Glyceraldehyde 3-phosphate dehydrogenase (GAPDH) antibody (Catalog number 60004-1-Ig, Proteintech, Rosemont, IL). After overnight incubation, the membranes were incubated with working dilutions of secondary antibodies specific to the primary antibody species (Catalog number 7074, Cell Signaling, Danvers, MA; Catalog number m-IgG sc-516102, Santa Cruz Biotechnology, Santa Cruz, CA), the membranes were then incubated in enhanced chemiluminescence (ECL) western blotting detection reagent (Amersham, Little Chalfont, United Kingdom) and imaged using a ChemiDoc imaging system (Bio-Rad, Hercules, CA).

Quantitative analysis

ImageJ software (National Institutes of Health, Bethesda, MD) was used to analyze the western blot images for pixel intensity. Utilizing GAPDH as a loading control, the ratio between pixel intensities of the protein of interest to GAPDH was acquired from the same membrane. The average ratio of three experimental repeats for each protein of interest was calculated. Comparisons of the average ratios were made using a two-tailed Student’s unpaired T-test, with a comparison being considered statistically significant at p ≤ 0.05.

## Results

**Figure 1:**
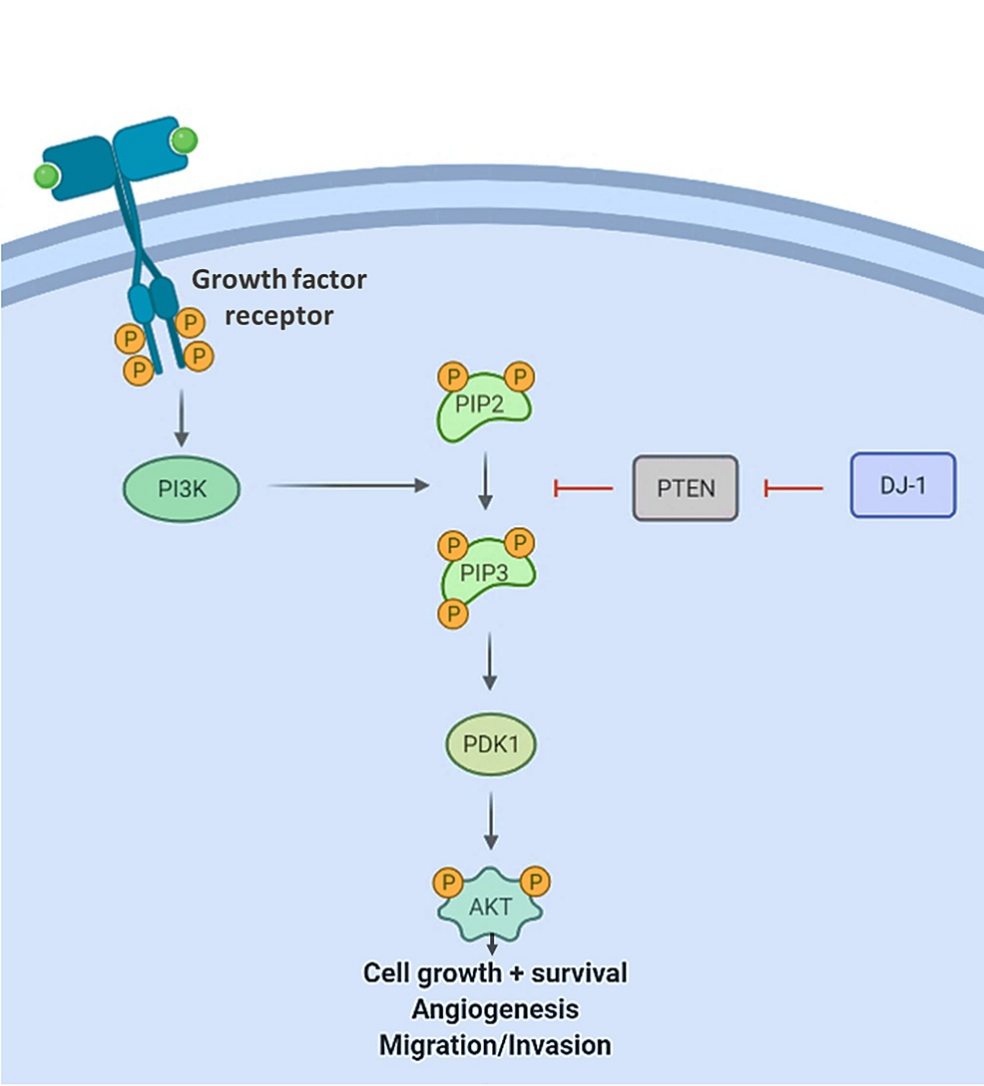
The involvement of PI3K/Akt pathway in cancer cell survival, proliferation, invasion, and migration. Schematic graph depicting the regulatory roles of PTEN and DJ-1 in the PI3K/Akt pathway. This pathway ultimately leads to the activation of the Akt kinase that modulates cancer cell growth, survival, invasion, and migration. This original figure was drawn by authors using Microsoft Office PowerPoint (Redmond, USA) and BioRender software.

This study examined the expression patterns of DJ-1, PTEN, and p-Akt in the progression of OSCC using an in-vitro cell model and found several unique trends about each protein. These three proteins were selected for their regulatory roles in the PI3K/Akt pathway involved in cancer cell survival, proliferation, invasion, and migration (Figure 1).

**Figure 2:**
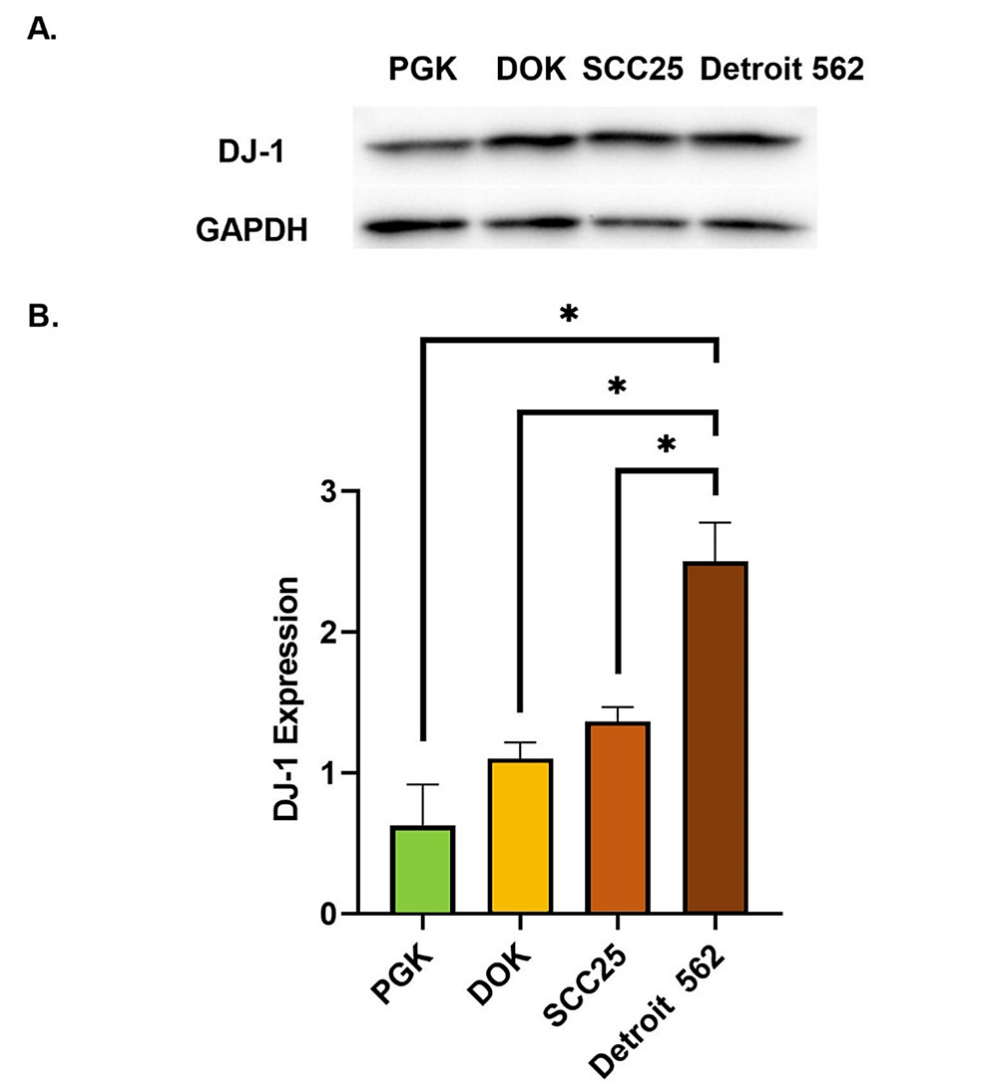
Quantitative analysis of DJ-1 expression in the four cell lines representing the successive steps of oral squamous cell carcinoma progression. (A) Western blot showing expression of DJ-1 and the GAPDH loading control in the four cell lines tested. (B) Comparison of DJ-1 expression relative to GAPDH expression in normal oral keratinocytes (PGK), dysplastic oral keratinocytes (DOK), locally invasive oral squamous cell carcinoma cells (SCC25), and metastatic oral squamous cell carcinoma cells (Detroit 562). Error bars represent standard error of the mean (SEM), * = p < 0.05.

DJ-1 expression was found to be upregulated in progressively greater amounts across the cell lines representing the progression steps of OSCC. The average DJ-1/GAPDH ratios were 0.63, 1.1, 1.36, and 2.5 for primary gingival keratinocytes (PGK), dysplastic oral keratinocytes (DOK), locally invasive SCC25, metastatic Detroit 562, respectively. Statistical analysis showed that DJ-1 expression was upregulated by about four-fold in the metastatic Detroit 562 cell line when compared to normal PGK (p = 0.01). DJ-1 expression was also found to be 2.3-fold greater in the metastatic Detroit 562 cells compared to the dysplastic DOK cells (p = 0.01). DJ-1 expression was markedly upregulated by about 1.8-fold in the metastatic cells, Detroit 562, when compared to the locally invasive SCC25 cells (p = 0.02) (Figure 2).

**Figure 3:**
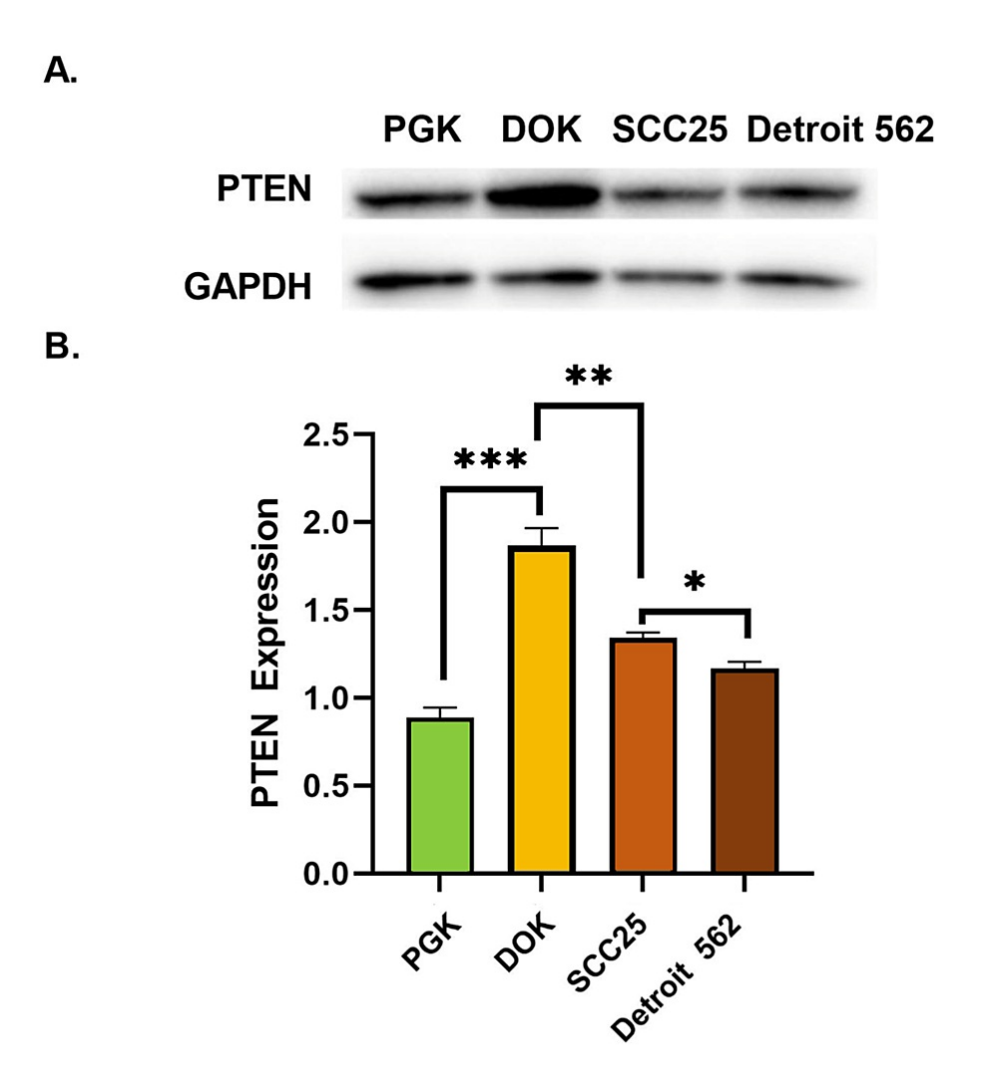
Quantitative analysis of PTEN expression in the four cell lines representing the successive steps of oral squamous cell carcinoma progression. (A) Western blot showing expression of PTEN and the GAPDH loading control in the four cell lines tested. (B) Comparison of PTEN expression relative to GAPDH expression in normal oral keratinocytes (PGK), dysplastic oral keratinocytes (DOK), locally invasive oral squamous cell carcinoma cells (SCC25), and metastatic oral squamous cell carcinoma cells (Detroit 562). Error bars represent standard error of the mean (SEM), * = p < 0.05, ** = p < 0.01, *** = p < 0.001.

**Figure 4:**
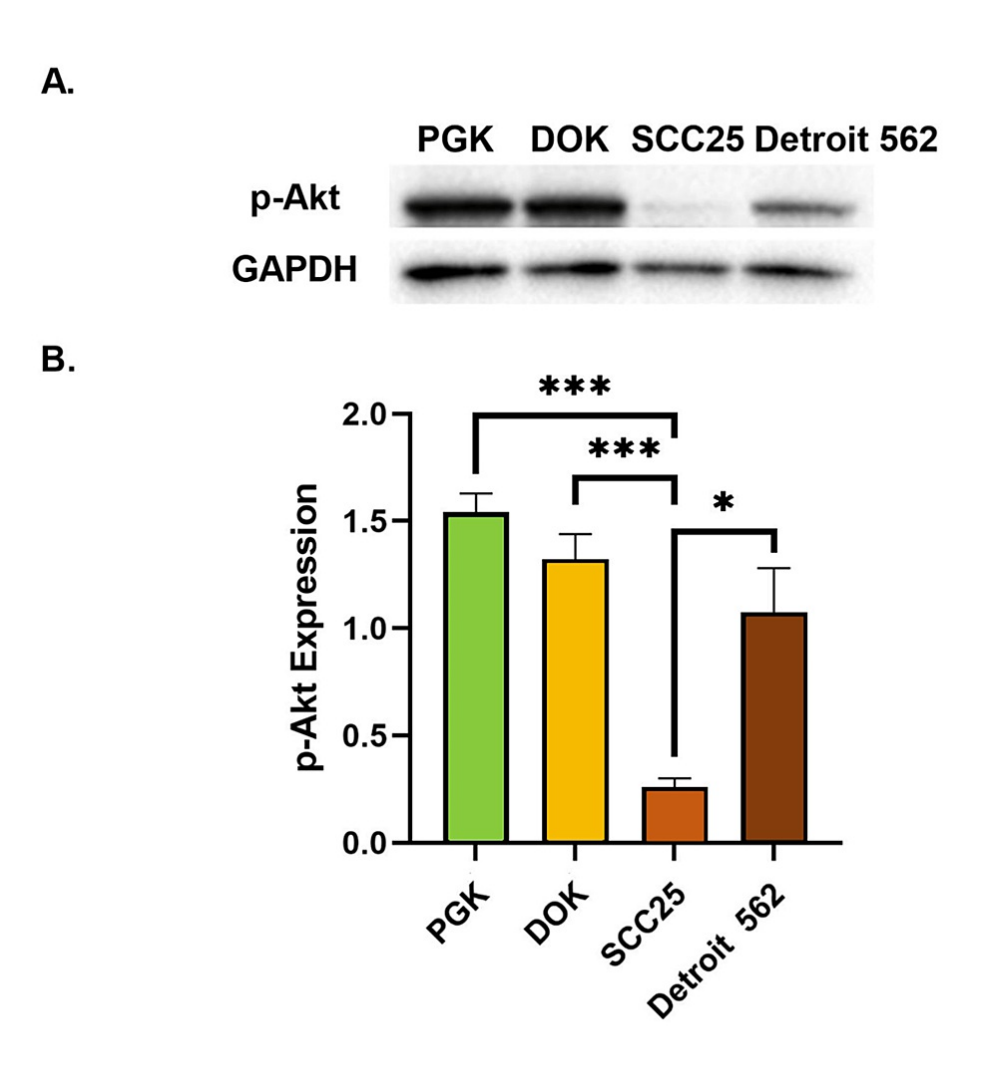
Quantitative analysis of p-Akt expression in the four cell lines representing the successive steps of oral squamous cell carcinoma progression. (A) Western blot showing expression of p-Akt and the GAPDH loading control in the four cell lines tested. (B) Comparison of p-Akt expression relative to GAPDH expression in normal oral keratinocytes (PGK), dysplastic oral keratinocytes (DOK), locally invasive oral squamous cell carcinoma cells (SCC25), and metastatic oral squamous cell carcinoma cells (Detroit 562). Error bars represent standard error of the mean (SEM), * = p < 0.05, *** = p < 0.001.

PTEN showed an increase in expression between the normal and dysplastic cell lines, while a gradual decrease was detected from the dysplastic cell line onwards alongside the successive progression steps. The average PTEN/GAPDH ratios were 0.89, 1.87, 1.34, and 1.17 in the PGK, DOK, SCC25, and Detroit 562 cell lines, respectively. All cell-to-cell comparisons were statistically significant, with a two-fold increase in PTEN expression in dysplastic DOK cells compared to normal keratinocytes (p = 0.001). A comparison of dysplastic DOK cells to locally invasive SCC25 cells showed a 1.4-fold decrease of PTEN in the SCC25 cells (p = 0.007). A 1.6-fold decrease in PTEN expression was found when comparing dysplastic DOK cells with metastatic Detroit 562 cells (p = 0.003). A comparison of the locally invasive SCC25 cells with metastatic Detroit 562 cells showed a 1.14-fold decrease in PTEN expression (p = 0.02) (Figure 3).

p-Akt was downregulated in the locally invasive SCC25 cell line in comparison to the normal keratinocytes. However, from the SCC25 line to the metastatic Detroit 562 cell line, an increase in p-Akt expression was observed. The average p-Akt/GAPDH ratios were 1.54, 1.32, 0.26, and 1.08 in the PGK, DOK, SCC25, and Detroit 562 cell lines, respectively. Interestingly, p-Akt expression was six-fold decreased in the locally invasive SCC25 cell line compared to normal keratinocytes (p = 0.0002). A comparison of the locally invasive SCC25 to dysplastic DOK cells showed a five-fold decrease in p-Akt expression (p = 0.001). Importantly, a four-fold increase in p-Akt expression was observed in the metastatic Detroit 562 cells when compared to the locally invasive SCC25 cells (p = 0.02) (Figure 4).

## Discussion

The biomarkers analyzed in this study were evaluated for their potential to complement the current methods of evaluating OSCC prognosis. The identification of novel biomarkers in the progression of OSCC would ultimately allow the utilization of a panel of biomarkers as a screening tool in prognosticating cancer and guiding treatment options.

DJ-1 is an oncogene that has been described as being mitogen dependent and is part of the Ras signal transduction pathway [14]. It is known to be involved in the proliferation and metastasis of certain cancers and has been shown to be an indicator of poor prognosis in glottic squamous cell carcinoma [10]. The present study found that DJ-1 expression quantitatively increases with successive steps of OSCC progression. Importantly, DJ-1 expression was markedly increased in the metastatic cancerous cells compared to all other cell types, consistent with a previous study suggesting a relationship between DJ-1 expression and lymph node metastases in supraglottic squamous cell carcinoma patients [12]. This highlights the potential utility of this biomarker to predict the metastatic potential of a certain tumor. In showing that DJ-1 can reliably distinguish between locally invasive and metastatic oral cancer, this study underscores the potential utility of DJ-1 as a prognostic biomarker for OSCC.

PTEN is a tumor suppressor involved in the regulation of the PI3K/Akt signaling pathway. PTEN loss or downregulation has been associated with various cancers [15,16]. It has been shown that DJ-1 can directly bind to PTEN and inhibit its phosphatase activity [24]. This study showed that PTEN levels gradually decreased from the dysplastic to cancerous forms, which is consistent with its role as a tumor suppressor gene product. Interestingly, PTEN was found to be elevated in dysplastic DOK cells compared to normal oral keratinocytes. This increase in PTEN expression from normal to dysplasia might reflect a cellular response to increased genetic instability. Considering its role as a tumor suppressor, a possible explanation might be that keratinocytes upregulate PTEN expression in response to transformative events and the accumulation of different mutations as an attempt to halt the progression through the cell cycle and prevent uncontrollable cell division. The gradual downregulation of PTEN expression after the dysplastic step is consistent with a predicted downward trend in PTEN expression along the successive steps of OSCC progression.

Akt, a Pleckstrin homology domain-containing kinase, also known as protein kinase B, is part of the PI3K/Akt pathway and is activated by phosphorylation into p-Akt [19,23]. p-Akt is involved in promoting increased survival, proliferation, and motility of cancer cells. Over expression of p-Akt is found to be associated with poor prognosis of several types of cancer [20,21,22,23,25]. This study found that there was a progressive decrease in p-Akt from normal to dysplastic DOK to locally invasive SCC25 cells. As this was an unexpected finding, it may point to other regulators that may be involved in the control of cell growth and proliferation in oral cancers. However, the increase in p-Akt that was observed in the metastatic Detroit 562 cell lines in comparison to the locally invasive SCC25 is consistent with the role of p-Akt as a promoter of motility and, thus, metastasis in the progression of oral cancer. A previous study found that there was increased p-Akt expression in esophageal squamous cell carcinoma (ESCC) samples with lymph node metastasis when compared with primary cancer [26].

This study relied on established human cell lines as representatives of the successive steps of tumor progression because of their rapid growth rate and relative homogeneity. However, cell lines may not reflect the same molecular changes that occur in human tumors since these cells do not account for the effects of the tumor microenvironment and the interactions with stromal, endothelial, inflammatory, and immune cells. Moreover, they do not represent the cellular heterogeneity within tumors. Therefore, in the next phase of our investigations, we aim to perform immunohistochemistry staining of DJ-1, PTEN, and p-Akt in normal, inflamed, dysplastic, and malignant oral tissue samples.

The findings of this pilot study warrant further clinical follow-up investigations to assess the relationship between these three related proteins, relative survival rates, and relapse rates in patient cohorts. The long-term goal of these investigations is to establish the utility of these molecular biomarkers in the clinical setting to supplement the current histopathological methods of assessment and prognosis.

## Conclusions

This study has shown significant quantitative differences in the expression of DJ-1, PTEN, and p-Akt amongst the different stages of OSCC development and progression, which paves the way for utilizing these proteins as prognostic biomarkers to predict outcomes and potentially serving as therapeutic targets for patients suffering from oral cancer.
